# Predator Dormancy is a Stable Adaptive Strategy due to Parrondo's Paradox

**DOI:** 10.1002/advs.201901559

**Published:** 2019-12-12

**Authors:** Zhi‐Xuan Tan, Jin Ming Koh, Eugene V. Koonin, Kang Hao Cheong

**Affiliations:** ^1^ Science and Math Cluster Singapore University of Technology and Design (SUTD) Singapore S487372 Singapore; ^2^ National Center for Biotechnology Information National Library of Medicine National Institutes of Health Bethesda MD 20894 USA; ^3^ SUTD‐Massachusetts Institute of Technology International Design Centre Singapore S487372 Singapore

**Keywords:** evolutionary dynamics, game theory, Parrondo's paradox, population dynamics, predatory–prey, predator dormancy

## Abstract

Many predators produce dormant offspring to escape harsh environmental conditions, but the evolutionary stability of this adaptation has not been fully explored. Like seed banks in plants, dormancy provides a stable competitive advantage when seasonal variations occur, because the persistence of dormant forms under harsh conditions compensates for the increased cost of producing dormant offspring. However, dormancy also exists in environments with minimal abiotic variation—an observation not accounted for by existing theory. Here it is demonstrated that dormancy can out‐compete perennial activity under conditions of extensive prey density fluctuation caused by overpredation. It is shown that at a critical level of prey density fluctuations, dormancy becomes an evolutionarily stable strategy. This is interpreted as a manifestation of Parrondo's paradox: although neither the active nor dormant forms of a dormancy‐capable predator can individually out‐compete a perennially active predator, alternating between these two losing strategies can paradoxically result in a winning strategy. Parrondo's paradox may thus explain the widespread success of quiescent behavioral strategies such as dormancy, suggesting that dormancy emerges as a natural evolutionary response to the self‐destructive tendencies of overpredation and related biological phenomena.

## Graphical abstract

Many predators produce dormant offspring to escape harsh conditions, but only at the cost of less offspring. The evolutionary stability of this costly dormancy is explained by Parrondo's paradox. Although both active and dormant forms of dormancy‐capable predators individually lose to perennially active predators, alternating between these losing strategies ensures better recovery after overpredation, resulting in a strategy that wins.

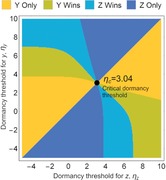

## 1 Introduction

The induction of dormancy in predatory species is a widespread adaptation that allows predators to survive harsh environmental conditions. Hibernation in mammals is common in temperate climes, and is considered to be an important factor in reducing their extinction risk.^1^ Diapause, a form of dormancy characterized by an obligatory delay in an organism's development, has been widely observed in insects and zooplankton.^2^, ^3^ Similar to the role of seed banks in plant ecology, diapause results in the creation of “biotic reservoirs” or “egg banks” that allow populations to disperse over time and tide over unfavorable periods such as winter or drought.^3^, ^4^, ^5^

While the evolutionary stability of this strategy is well‐established in the case of temporally varying environments,^3^, ^4^, ^6^, ^7^ dormancy has also been observed in environments with minimal abiotic fluctuations, such as diapausing zooplankton in large tropical lakes.^3^, ^8^, ^9^ Given that entering dormancy is costly, whether due to the energy required to produce hardy resting eggs or the inability to forage while dormant, this finding leads to a striking conundrum: how does costly dormancy persist and remain competitive as an evolutionary strategy even in environments with minimal abiotic variation over time?

Prior work has suggested that dormancy is advantageous in nonseasonal environments because it reduces the population fluctuations caused by overpredation, thereby reducing extinction risk as well. As found experimentally by McCauley et al.^10^ and analyzed theoretically by Kuwamura et al.,^11^ predator dormancy can suppress the emergence of large‐amplitude cycles in predator and prey populations. These findings are proposed to explain the nonoccurrence of the paradox of enrichment—the classic prediction that enriched resource levels counter‐intuitively cause large‐amplitude fluctuations that increase extinction risk.^12^ Dormancy is thought to be competitive because it can prevent such large‐amplitude cycles from occurring.

However, this explanation is inadequate as an account for both the competitive advantage and evolutionary stability of dormancy. First, it remains a matter of debate as to whether large‐amplitude population cycles significantly increase extinction risk,^13^ a connection that must exist if preventing such cycles is to provide an evolutionary advantage. Second, the competitive exclusion principle suggests that when population cycles are suppressed, dormancy‐practicing predators—henceforth referred to as dormitive predators—should be driven to extinction by perennially active mutants.^14^, ^15^, ^16^ If prey populations stay mostly constant, rather than cycling through low levels, then as long as entering dormancy costs energy and prevents predation, dormancy should never be advantageous relative to staying active and consuming more prey. It is only when prey levels are occasionally low that dormancy might be useful. Preventing population cycles thus cannot confer dormitive predators a competitive edge over perennially active mutants.

What other mechanism might explain the competitiveness of dormancy in the absence of abiotic changes? Recent theoretical work has shown that environmentally destructive species can survive by alternating between “nomadic” and “colonial” forms of behavior.^17^ Similar to predators which alternate between active and dormant forms, these nomadic‐colonial populations can either undergo rapid but destructive growth as “colonists,” or allow their habitat to recuperate as “nomads” while suffering population decay. Each behavior leads to extinction on its own, but the population is able to survive by alternating between these individually maladaptive behaviors. The population thus exhibits Parrondo's paradox, an abstraction of the phenomenon of flashing Brownian ratchets^18^, ^19^, ^20^ in which alternating between two losing strategies produces a strategy that wins.^21^, ^22^, ^23^, ^24^ Furthermore, unlike other ecological manifestations of the paradox,^25^, ^26^ the success of nomadic‐colonial alternation does not require any exogenous environmental variation. The setting is thus analogous to the case of predator dormancy in the absence of abiotic variation.

Motivated by this theoretical analysis, we develop and study a population model in which a dormitive predator competes with a perennially active predator over the same prey population. In game‐theoretic terms, both the dormant and active forms of a dormitive predator are losing strategies relative to a perennially active counterpart. The dormant form loses because it can only decay in population, not grow, while the active form loses because it expends extra energy on producing dormant descendants, instead of hunting for prey. Yet Parrondo's paradox suggests that alternating between these two losing strategies might yield a winning strategy. By carefully exploring the parameter space of our model, we investigate whether and when this counter‐intuitive advantage of predator dormancy can arise. We further investigate whether dormancy is an evolutionarily stable strategy^27^, ^28^, ^29^—i.e., whether dormitive predators are resistant to invasion by mutant strains that either do not practice dormancy, or that are “less dormant,” in that they practice dormancy only under more extreme conditions.

## 2 Results

### 2.1 Population Model

We define a model that introduces an additional predator population to an existing model of predator dormancy,^11^ which itself is adapted from the Lotka–Volterra‐derived model of Rosenzweig and MacArthur.^30^ In this model, two predator populations, *y* and *z*, compete over a prey population, *p*. *y* is the density of active predators, and *z* = *z*_1_ + *z*_2_ is the density of the dormitive predators, with *z*_1_ and *z*_2_ corresponding to the active and dormant subpopulations, respectively. The rate equations are

(1a) $$\dot{p}=r\left(1-\frac{p}{K}\right)p\;\;-f_{y}\left(p\right)y\;\;-f_{z}\left(p\right)z_{1}$$

(1b) $$\dot{y}=k_{y}f_{y}\left(p\right)y-d_{y}y$$

(1c) $${\dot{z}}_{1}=k_{z_{1}}\mu \left(p\right)f_{z}\left(p\right)z_{1}+\alpha z_{2}-d_{z_{1}}z_{1}$$

(1d) $${\dot{z}}_{2}=k_{z_{2}}\left(1-\mu \left(p\right)\right)f_{z}\left(p\right)z_{1}-\alpha z_{2}-d_{z_{2}}z_{2}$$

Parameter and function descriptions can be found in **Table** **1** alongside reference units and default simulation values. For illustration purposes, we use values drawn from the plankton modelling literature.^31^, ^32^, ^33^ Conceptually, the prey population undergoes logistic growth with carrying capacity *K*, as well as predation from *y* and *z*_1_ at rates *f*_*y*_(*p*) and *f*_*z*_(*p*), respectively. The active predator *y* grows in proportion to the predation rate *f*_*y*_(*p*) and growth efficiency *k*_*y*_, while dying at rate *d*_*y*_. The active form of the dormitive predator *z*_1_ behaves similarly, except that (i) it is supplemented by dormant predators *z*_2_ returning to the active form at the “hatching rate” α; (ii) only a fraction of the energy gained from predation, μ(*p*), is used to produce active progeny of the dormitive predator. The remaining fraction, (1 − μ(*p*)), is used to produce dormant progeny of the predator, *z*_2_.

**Table 1:** Population, parameter, and function descriptions, along with reference units and default simulation values (chosen to fall within the range of plankton ecosystems^31^, ^32^, ^33^). Quantities without units listed are dimensionless

Population	Description		Units
*p*	Prey density		mg L^−1^
*y*	Active predator density		mg L^−1^
*z*_1_	Dormitive predator (active form) density		mg L^−1^
*z*_2_	Dormitive predator (dormant form) density		mg L^−1^
Parameter	Description	Values	Units
*r*	Prey growth rate	0.50	day^−1^
*K*	Prey carrying capacity	4–25	mg L^−1^
$k_{y},k_{z_{1}}$	Predator growth efficiency (active)	0.50	–
$k_{z_{2}}$	Predator growth efficiency (dormant)	0.25	–
$d_{y},d_{z_{1}}$	Predator death rate (active)	0.25	day^−1^
$d_{z_{2}}$	Predator death rate (dormant)	0.05	day^−1^
*c*_*y*_, *c*_*z*_	Predator foraging efficiencies	0.40	day^−1^ mg^−1^ L
*h*_*y*_, *h*_*z*_	Predator handling times	0.75	day
α	Dormancy termination rate	0.05	day^−1^
η	Dormancy switching threshold	2.5	mg L^−1^
σ	Dormancy switching width	1.0	mg L^−1^
Function	Description		Units
*f*_*y*_(*p*), *f*_*z*_(*p*)	Functional responses (predation rates)		day^−1^
μ(*p*)	Dormancy switching function		–

Rates of predation are modelled by monotonically increasing functional responses *f*_*y*_(*p*) and *f*_*z*_(*p*). Specifically, Holling type II functional responses^30^ are used

(2) $$f_{q}\left(p\right)=\frac{c_{q}p}{1+c_{q}h_{q}p},\;q\in \left\{y,z\right\}$$

where *c* is the foraging efficiency and *h* is the handling time the predator requires to process each item of prey. Hence, the response is linear (*f*(*p*) ≃ *cp*) when *p* is small, but converges to a fixed rate *f*(*p*) = 1/*h* as *p* → ∞.

### 2.2 Modeling Predator Dormancy

**Figure 1:**
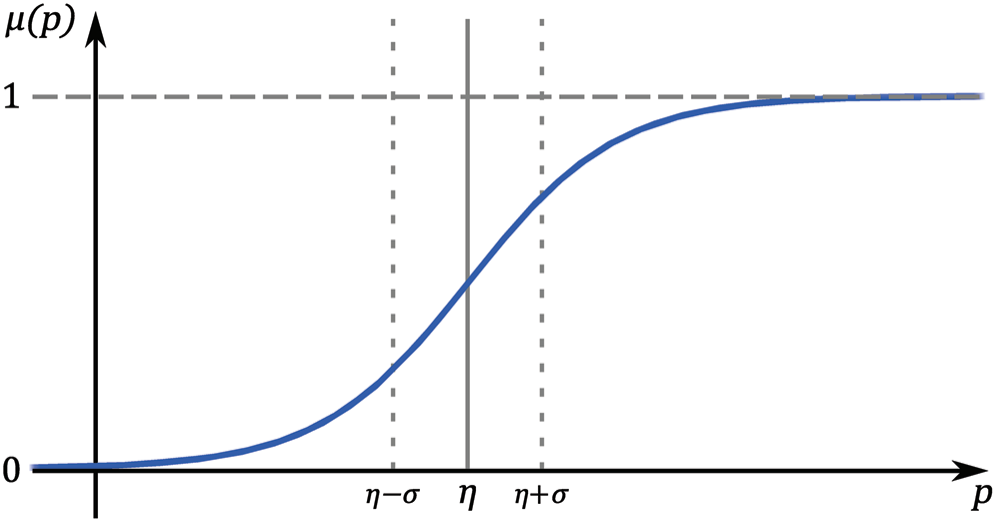
We model the fraction of energy used to produce active progeny as a sigmoid switching function, μ(*p*). η is the switching threshold, and σ controls the sharpness of switching. In high‐prey conditions (*p* − η → ∞), effectively all offspring are active (μ(*p*) → 1), but in low‐prey conditions (*p* − η → −∞), effectively all offspring are dormant (μ(*p*) → 0).

In the absence of abiotic variation, dormancy in many species can still be induced by the absence of prey.^3^, ^34^ Following the analysis by Kuwamura et al.,^11^ we model the induction of dormancy as a sigmoid switching function μ of the prey density *p*

(3) $$\mu \left(p\right)={\left[1+\exp\left(-\frac{p-\eta }{\sigma }\right)\right]}^{-1}$$

where η is the switching threshold such that μ(*p*) > 1/2 whenever *p* > η, and σ is a width parameter that controls the sharpness of switching, with smaller values resulting in sharper switching. **Figure** **1** shows a diagram of this switching function.

We are interested in exploring the case where dormancy is costly, both in terms of energy and opportunity. Dormancy always comes with some opportunity cost, but if entering dormancy were energetically cheaper than remaining active (for instance in mammalian hibernation, or in the unlikely case that resting eggs are easier to produce than subitaneous ones), then it would be unsurprising to find conditions in which dormancy gives an advantage. Thus, we restrict the parameters such that it is easier to produce active offspring than dormant offspring $\left(k_{z_{1}}>\;\;k_{z_{2}}\right)$, as in, for instance, diapausing zooplankton.^3^

Because we are also interested in studying how a dormitive predator might out‐compete an active predator through dormancy alone, we further assume that the active predator *y* is at least as proficient as the active form *z*_1_ of the dormitive predator. More precisely, we assume that the active predator captures and consumes prey at least as efficiently, and that it dies at most as rapidly, as the dormitive predator (*f*_*y*_(*p*) ≥ *f*_*z*_(*p*), $k_{y}\geq k_{z_{1}}$, $d_{y}\leq d_{z_{1}}$). Finally, we assume that the dormitive predator has a lower death rate in dormancy than in activity $\left(d_{z_{1}}>d_{z_{2}}\right)$. Otherwise, even in the absence of an active competitor, there would be no survival advantage to dormancy.

### 2.3 Absence of Population Cycles Results in Extinction of Dormancy

The competitive exclusion principle implies that in a regime of population stability where population cycles are suppressed, the dormitive predator *z* will be driven to extinction by the active predator *y*. More precisely, this regime of population stability refers to the range of parameters under which the system converges to a stable fixed point.

Apart from the trivial fixed points $x_{0}^{*}=\left(0,0,0,0\right)$ (no prey or predators) and $x_{p}^{*}=\left(K,0,0,0\right)$ (prey at carrying capacity), there are only two other fixed points of interest: the active‐only equilibrium $x_{y}^{*}=\left(p_{y}^{*},y^{*},0,0\right)$, and the dormitive‐only equilibrium $x_{z}^{*}=\left(p_{z}^{*},0,z_{1}^{*},z_{2}^{*}\right)$. As predicted by the competitive exclusion principle, when dormancy is costly, we found analytically that there is no fixed point $x_{yz}^{*}=\left(p^{†},y^{†},z_{1}^{†},z_{2}^{†}\right)$ where both predators coexist, indicating that stable predator coexistence is impossible. We further found that adding even a small number of active predators *y* destabilizes the population and pushes it away from the dormitive‐only equilibrium $x_{z}^{*}$. Thus, dormitive predators cannot suppress the emergence of active mutants. On the other hand, we found that the active‐only equilibrium $x_{y}^{*}$ is stable to increases in *z*_1_ and *z*_2_. As long as $x_{y}^{*}$ is also stable to changes in *p* and *y*, a system that diverges from $x_{z}^{*}$ will converge to $x_{y}^{*}$—that is, the emergence of active mutants eventually drives the dormitive population to extinction. (Derivations can be found in the Experimental Section.)

**Figure 2:**
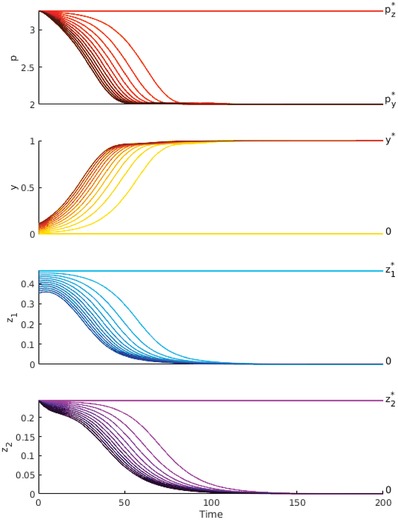
The competitive exclusion of dormancy in an environment that suppresses population cycles. Carrying capacity *K* = 4, other parameters chosen as in Table 1. The system is initialized at the dormitive‐only equilibrium $x_{z}^{*}$, except with a small *y*‐perturbation $\Delta y\in [0,z_{1}^{*}/4]$ subtracted from *z*_1_ and added to *y*, thereby simulating a dormitive *z* population invaded by active *y* mutants. Darker lines correspond to initial conditions with more active mutants.

This process of competitive exclusion is clearly depicted in **Figure** **2** across a range of initial population levels. The initial conditions are chosen such that $p=p_{z}^{*}$ and $y+z_{1}=z_{1}^{*}$, while *y* is varied from just above zero to $z_{1}^{*}/4$. This simulates a system that is originally at the dormitive‐only equilibrium $x_{z}^{*}$, which then experiences a mutation event at *t* = 0 where some fraction of the dormitive predators lose the dormancy trait. The dormitive predators and the active mutants are of equal proficiency ( *f*_*y*_ = *f*_*z*_, $k_{y}=k_{z_{1}}$, $d_{y}=d_{z_{1}}$), so the only difference is that the dormitive predators divert some energy toward producing dormant offspring *z*_2_. Even in cases where *y* is initially very small, the system converges to the active‐only equilibrium $x_{y}^{*}$, demonstrating that predator dormancy is an evolutionarily unstable trait when population cycles are absent.

### 2.4 Large‐Amplitude Population Cycles Allow Dormancy to Flourish

Because dormancy is driven to extinction in the regime where population cycles are suppressed, we surmise that dormancy might be competitive only outside of this regime. In predator–prey models, population cycles emerge spontaneously when the carrying capacity of the prey, *K*, exceeds a critical level—i.e., the system undergoes a Hopf bifurcation.^35^, ^36^ This phenomenon is also called the paradox of enrichment, because an increase in resource levels (*K*) counter‐intuitively leads to large‐amplitude population cycles that might increase extinction risk.^12^, ^31^ However, such “risky” population cycles are precisely what might give dormancy an advantage, because it is only when the system cycles through low levels of prey that a predator which enters dormancy might fare better than a perennially active predator. It is thus natural to consider the regime where this Hopf bifurcation has occurred to investigate if dormancy can survive competition.

**Figure 3:**
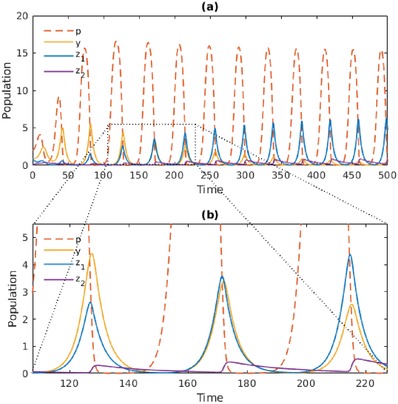
At *K* = 18, high‐amplitude population cycles occur, enabling the dominance of dormitive predators over active predators. a) *y* and *z*_1_ converge to a limit cycle where *z*_1_ > *y*. b) The dynamics are magnified, showing how *z*_1_ recovers more rapidly from prey depletion by using *z*_2_ as a reservoir. Parameters take the values in Table 1 except *K* = 18, $d_{z_{2}}=0.025$, and α = 0.025.

In a system with only active predators, we term the carrying capacity at which the Hopf bifurcation occurs as $K_{y}^{h}$. Above this critical value, the active‐only equilibrium $x_{y}^{*}$ loses its stability, and a stable limit cycle replaces the equilibrium. In a system with only dormitive predators, we term the corresponding bifurcation point as $K_{z}^{h}$. Expressions for $K_{y}^{h}$ and $K_{z}^{h}$ are given in the Experimental Section. Because our system has both active and dormitive predators, it is appropriate to examine the regime where *K* exceeds both $K_{y}^{h}$ and $K_{z}^{h}$. When *K* exceeds both of these values only slightly, we found that the dormitive predator is still driven to extinction. However, a sufficiently high value of *K* results in a large amplitude limit cycle where the dormitive predator *z*_1_ not only coexists with, but eventually dominates the active predator *y* in population size (**Figure** **3**a).

The area within the dotted lines in Figure 3a is magnified in Figure 3b. By closely examining the latter, we can understand the mechanism by which the dormitive predator out‐competes the active predator when *K* is high. The enriched environment causes large‐amplitude population cycles, resulting in recurrent collapses of the prey population. Each prey collapse is rapidly followed by collapses of the active predator, *y*, and the active form of the dormitive predator, *z*_1_. However, the low death rate of the hardy dormant population, *z*_2_, allows it to serve as a biotic reservoir for *z*_1_. This enables the dormitive species to recover more rapidly when prey populations start rising again, thereby dominating the active species in population size, despite the latter being a more efficient predator for a fixed level of prey. At low *K*, this recovery effect is not strong enough, such that the dormitive species still goes extinct. However, when *K* is sufficiently high, the extremity of fluctuations in the prey population drives the active species extinct, whereas the dormitive predator is able to survive by entering dormancy.

**Figure 4:**
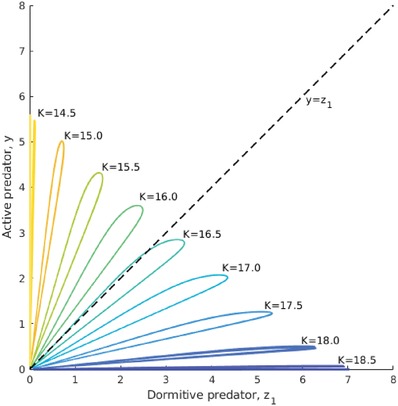
Limit cycle trajectories for different values of $K>K_{y}^{h},K_{z}^{h}$, plotted in *y*–*z*_1_ space. When *K* ≲ 14, *z*_1_ is extinct. When *K* ≳ 19, *y* is extinct. Between these values, the active and dormitive predators coexist. As *K* increases, the ratio of *z*_1_ to *y* increases, until eventually *z*_1_ > *y* throughout the entire limit cycle. Apart from *K*, parameters take the values in Table 1.

More generally, we found that as *K* increases, the dormitive population transitions from extinction, to coexistence with the active population, to driving the active predators extinct. This can be seen from the phase portraits in **Figure** **4**, depicting the limit cycles of *z*_1_ against *y* for multiple values of *K*. When *K* ≲ 14, the limit cycle forms a line along the vertical axis, indicating that only the active predator is present, whereas the dormitive predator is extinct. As *K* increases, the limit cycle extends horizontally, indicating a higher ratio of *z*_1_ to *y* as the population oscillates. The limit cycle eventually crosses the diagonal *z*_1_ = *y*, reflecting the dominance of *z*_1_ over *y* throughout the oscillation. Finally, for *K* ≳ 19, the limit cycle forms a line along the horizontal axis, indicating that only the dormitive predator survives. In other words, as the environment is enriched (*K* increases), the competitiveness of dormancy increases, eventually driving perennial activity extinct. Although Figures 3 and 4 only show this for the case of equally proficient dormitive and active predators (*f*_*y*_ = *f*_*z*_, $k_{y}=k_{z_{1}}$, $d_{y}=d_{z_{1}}$), our simulation results show that this remains the case when the perennially active predator is more proficient (Supporting Information).

### 2.5 Conditions for the Competitive Advantage of Dormancy

**Figure 5:**
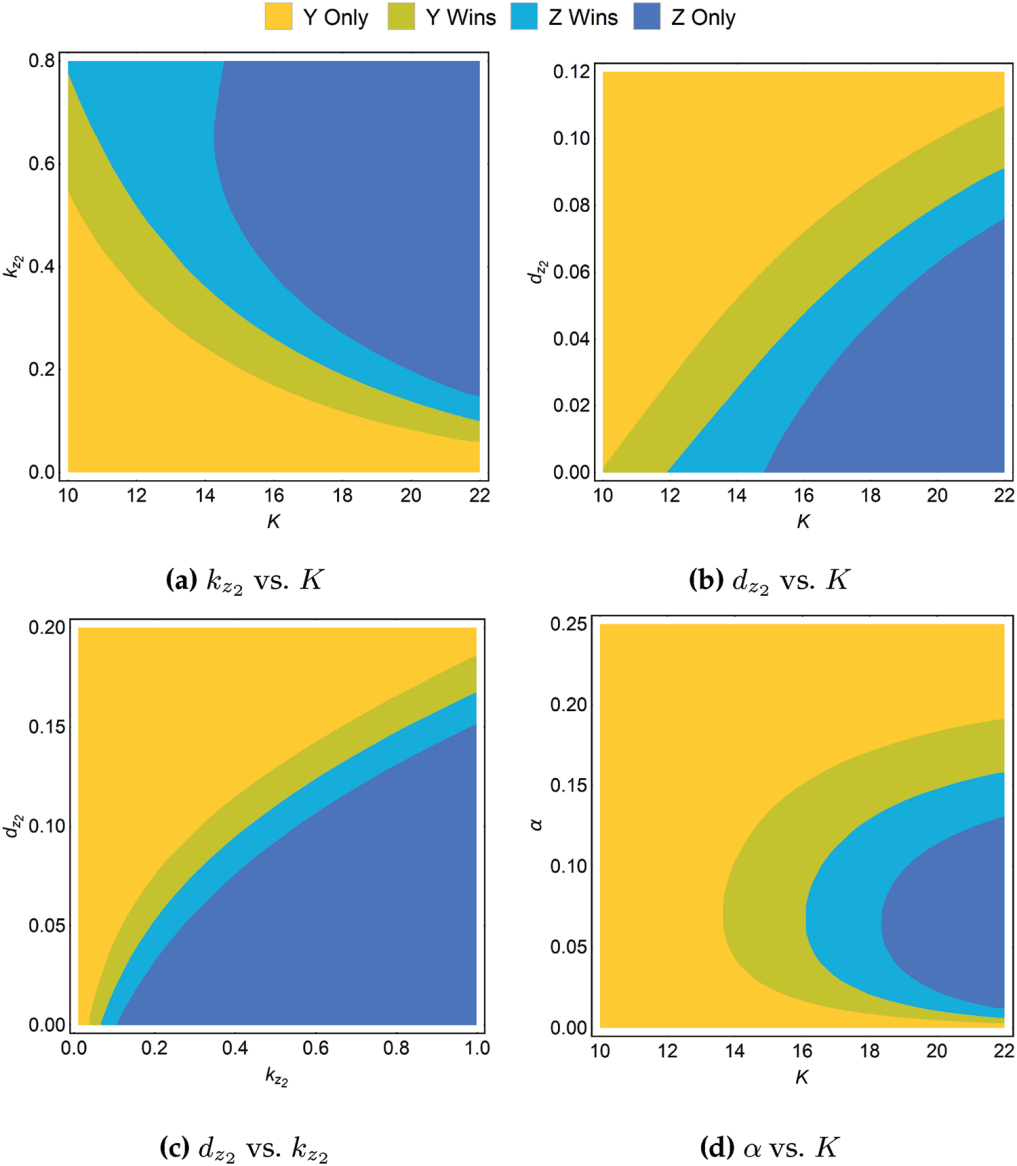
Phase diagrams showing the parameter regimes where only the active predator survives (*y* only), the active predator dominates the dormitive predator (*y* wins), the dormitive predator dominates the active predator (*z* wins), or only the dormitive predator survives (*z* only). In general, the dormitive predator has a stronger competitive advantage as *K* increases, $k_{z_{2}}$ increases, $d_{z_{2}}$ decreases, and when α is an intermediate range. Parameter values as in Table 1 unless stated otherwise.

Because dormancy confers a competitive advantage by accelerating recovery after a collapse of the prey population, several parameters can be deduced to affect the competitiveness of dormancy: the prey carrying capacity, *K*, the growth constant of the dormant form, $k_{z_{2}}$, the death constant of the dormant form, $d_{z_{2}}$, and the “hatching rate,” that is, the rate of transition from the dormant form to the active form, α. Keeping to the constraint that the active predator is at least as proficient as the dormitive predator, we performed simulations across a wide range of the aforementioned parameters. For each set of parameters, the dominance or extinction of the active and dormitive species was computed, where “dominance” refers to the long‐run average population level of one species being higher than that of the other. Details can be found in the Experimental Section. **Figure** **5** shows the resulting phase diagrams in the case where *y* and *z*_1_ are equally proficient, as in preceding analyses.

Each of the phase diagrams display trends that correspond intuitively to the mechanism by which dormancy confers an advantage. As already shown, increases in *K*, the prey carrying capacity, give an advantage to the dormitive predator *z*, while causing the active predator to “lose” and eventually go extinct (Figure 5a,b,d). This occurs because enriched resource levels cause increasingly drastic population fluctuations, disadvantaging the active predator. As the growth constant of the dormant form $k_{z_{2}}$ increases, or as the corresponding death rate $d_{z_{2}}$ decreases, this also gives the dormitive predator an advantage (Figure 5a–c), because such changes improve the ability of the dormant population to act as a biotic reservoir in the absence of prey.

The relationship between the “hatching rate,” α, and the competitiveness of dormancy is the most interesting because it is nonmonotonic, as can be observed in Figure 5d. Nonetheless, this too has an intuitive explanation. While higher values of α deplete the dormant population too rapidly for it to tide over harsh conditions, values of α that are too low can slow down population recovery, inhibiting the advantage of dormancy as a response to collapses of the prey population.

Collectively, we found that the competitive advantage of dormancy is promoted when the environment is enriched (higher *K*), when the dormant form grows more rapidly and dies more slowly (higher $k_{z_{2}}$ and lower $d_{z_{2}}$), and when the “hatching rate” α is within a favorable intermediate range. These trends continue to hold even when the active predator *y* is more proficient than the active form of the dormitive predator *z*_1_ (Supporting Information), suggesting that one predator can outcompete another more proficient predator in the same niche simply by practicing dormancy.

### 2.6 Evolutionary Stability of Threshold‐Induced Dormancy

Our results imply that dormancy is evolutionarily stable when *K* is sufficiently high. However, we have only compared two evolutionary strategies thus far: dormancy induced at fixed prey threshold η, and a complete absence of dormancy altogether. This approach neglects potential mutants which enter dormancy at a lower threshold. Such mutants might out‐compete the wild type, because they can exploit the prey left behind when the wild type enters dormancy at a higher threshold. To firmly establish the evolutionary stability of threshold‐induced dormancy, it is thus necessary to compare the relative success of dormitive predators with differing thresholds.

In order to do so, we modified our population model such that both predator species *y* and *z* are capable of dormancy, with active subpopulations *y*_1_, *z*_1_ and dormant subpopulations *y*_2_, *z*_2_, respectively. Each species has its own dormancy threshold, η_*y*_ and η_*z*_, with the corresponding sigmoid switching functions μ_*y*_, μ_*z*_. We keep all other parameters (e.g., growth constants and death rates) equal for both species. This is to investigate the case where mutations only affect the dormancy threshold, and thereby determine if an evolutionary stable threshold η_c_ exists. The modified model is described by the rate equations

(4a) $$\dot{p}=r\left(1-\frac{p}{K}\right)p-f\left(p\right)y_{1}-f\left(p\right)z_{1}$$

(4b) $${\dot{q}}_{1}=k_{1}\mu_{q}\left(p\right)f\left(p\right)q_{1}+\;\;\alpha q_{2}-\;\;d_{1}q_{1}$$

(4c) $${\dot{q}}_{2}=k_{2}\left(1-\mu_{q}\left(p\right)\right)f\left(p\right)q_{1}-\alpha q_{2}-d_{2}q_{2}$$

where *q* is a placeholder for either *y* or *z*.

**Figure 6:**
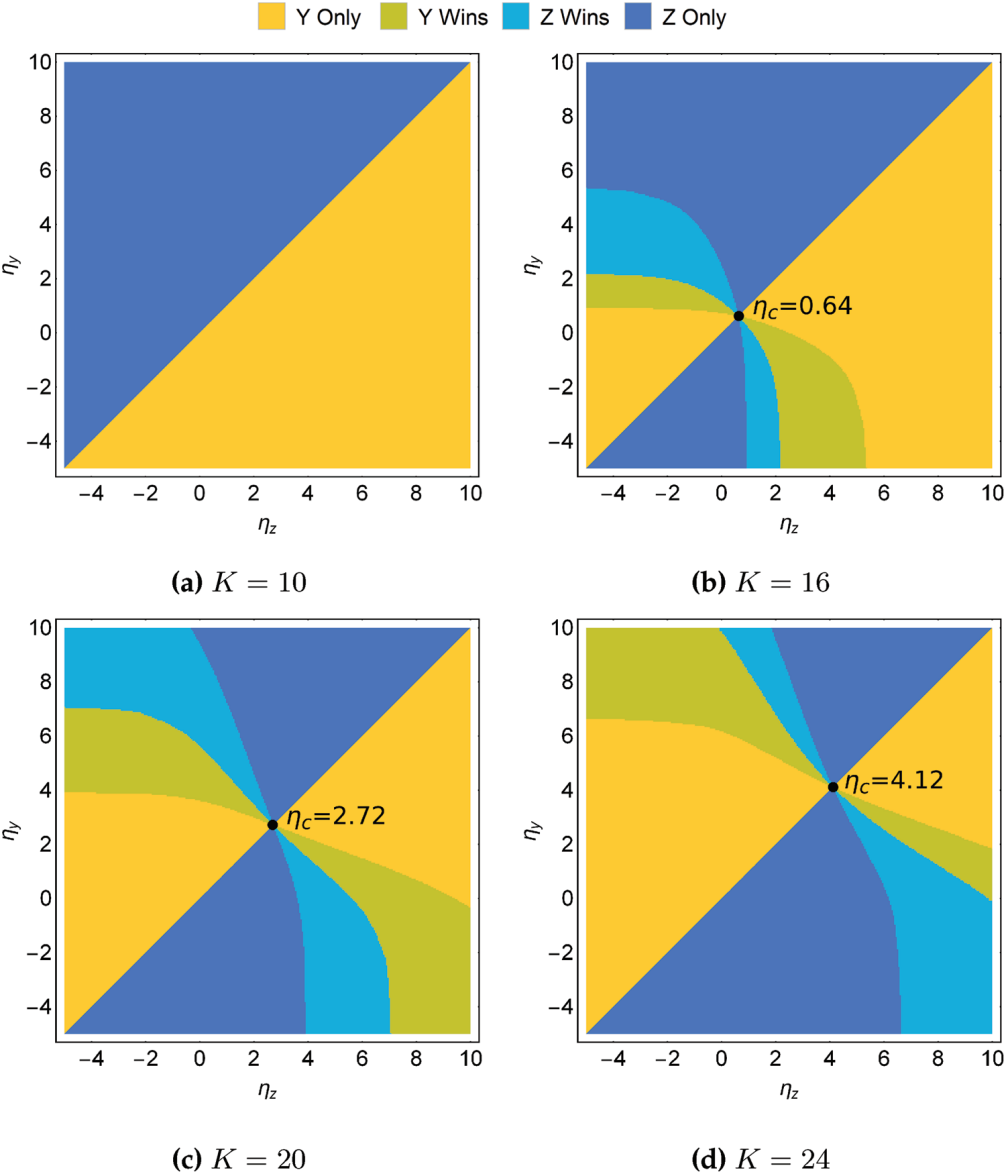
Pairwise invasibility plots comparing the evolutionary success of competing dormitive predators with differing dormancy thresholds η_*y*_ and η_*z*_. a) When *K* is low, population cycles are suppressed, and the predator with the higher dormancy threshold always goes extinct. b,c) When *K* is sufficiently high, large‐amplitude population cycles occur, and an evolutionary stable strategy, η_c_, emerges. When η_*y*_ = η_c_, and η_*z*_ ≠ η_c_, *z* loses to *y*, and vice versa. Parameter values as in Table 1 except for *K*.

With this modified model, we found analytically and confirmed through numerical simulations that coexistence of predators at a stable fixed point is impossible when their dormancy thresholds η_*y*_ and η_*z*_ differ (see the Experimental Section). The pairwise invasibility plot^37^ in **Figure** **6**a shows that when population cycles are suppressed at $K=10<\min\left(K_{y}^{h},K_{z}^{h}\right)$, a higher dormancy threshold η results in extinction. This is because a species with a lower dormancy threshold will exploit the prey forsaken by a higher‐threshold species, thereby out‐competing the latter. As such, both strains *y* and *z* will repeatedly evolve toward a lower η to out‐compete the other. Because η_*y*_ and η_*z*_ approach negative infinity, this means dormancy at any threshold is evolutionarily unstable, generalizing the results stated earlier when comparing dormitive and perennially active predators (Sections 2.3–2.5).

**Figure 7:**
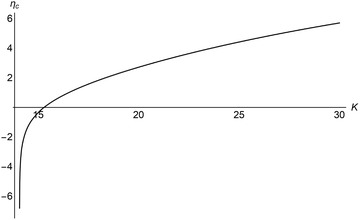
The evolutionarily stable threshold for dormancy, η_c_, increases with the prey carrying capacity, *K*. At *K* ≃ 14, there is a negative singularity, implying that dormancy is evolutionarily unstable below this point. As *K* increases, η_c_ first increases rapidly until *K* ≃ 15.5, then tapering off and increasing more linearly with *K*. Parameter values as in Table 1 unless stated otherwise.

In contrast, when *K* is above the Hopf bifurcation point, large‐amplitude population cycles emerge. As can be seen from the pairwise invasibility plots in Figure 6b–d, this creates an advantage for higher dormancy thresholds up to a point. For example, when *K* = 20 and η_*y*_ = −2 (Figure 6c), any predator *z* with −2 ≲ η_*z*_ ≲ 4 causes predator *y* to go extinct, and a threshold of up to η_*z*_ ≃ 7 still ensures that predator *z* is dominant. Indeed, the simulations demonstrate the emergence of an evolutionarily stable critical threshold, η_c_ (Figures 6 and 7). At η_*y*_ = η_*z*_ = η_c_, the four colored regions meet—any perturbation in η_*y*_ leads to predator *z* winning, whereas any perturbation in η_*z*_ leads to predator *y* winning. Furthermore, higher thresholds η_*y*_, η_*z*_ are favored when both are below η_c_, and conversely, lower thresholds η_*y*_, η_*z*_ are favored when both are above η_c_. This means that η_c_ corresponds to an evolutionarily stable strategy.

Figure 6b–d also indicates that η_c_ increases with *K*. To explore this relationship, we numerically solved for η_c_ over a range of values of *K* (**Figure** **7**) and showed that η_c_ indeed grows with *K*, rapidly increasing from η_c_ = −∞ when *K* ≲ 15.5, and then tapering off and increasing linearly. This nonlinear trend coheres with our earlier findings for a fixed value of η = 2.5. When $K<\min\left(K_{y}^{h},K_{z}^{h}\right)$, dormancy is evolutionarily unstable (Figure 2), equivalent to η_c_ = −∞. As *K* increases above $\max\left(K_{y}^{h},K_{z}^{h}\right)$, dormancy begins to have an increasing advantage against nondormancy (Figure 4), implying that the critical level η_c_ should increase as well. This effect can also be explained through the mechanism for the competitive advantage of dormancy. Higher *K* leads to more extreme population cycles, which then favor entering dormancy at a higher prey threshold η_c_ as an adaptation to the sharper collapses in prey levels.

## 3 Discussion and Conclusion

Our analysis of threshold‐induced predator dormancy has elucidated an intriguing mechanism for its adaptiveness and evolutionary stability even in the absence of seasonal abiotic variation. On the surface, at least two aspects of this mechanism may appear counter‐intuitive. The first is how the dormitive predator subverts the competitive exclusion principle, out‐competing perennially active predators that are just as proficient as the active form of the dormitive predator, if not more. The second is how, contrary to the intuition that dormancy should fare better in poor environments, the advantage of dormancy increases with the level of resource enrichment, as measured by the prey carrying capacity, *K*. Yet, more careful reasoning shows how these aspects can be consistently explained, thereby accounting for the otherwise surprising existence of predator dormancy in nonseasonal habitats.

The subversion of competitive exclusion can be understood as a case of Parrondo's paradox: two losing strategies, when combined, result in a strategy that wins. Neither the dormant form nor the active form of the dormitive predator is individually competitive against the perennially active predator. This is because the dormant form can only decrease in population on its own, while the active form diverts energy to the generation of dormant offspring instead of active offspring (μ(*p*) < 1 for any *p*), thereby losing out to predators which do not divert any energy to dormancy. However, through threshold‐induced alternation between these two strategies, a strategy that out‐competes perennial activity emerges.

So why does this counter‐intuitive result occur? Just as in other manifestations of Parrondo's paradox, one of the “losing” strategies is capital‐dependent (in the case of economic games) or density‐dependent (in the case of ecological games), while the other strategy is not.^17^, ^22^, ^23^, ^25^, ^26^, ^38^, ^39^, ^40^, ^41^, ^42^ In particular, the growth of the active form of the dormitive predator is dependent on the density of the prey, which itself depends on the density of the active predator. The dormant form, in contrast, has a density‐independent death rate. This entails the differential advantage of each strategy depending on the density conditions. Under low‐prey conditions, the active form dies rapidly, being inferior to the dormant form, which dies at a slow rate regardless of prey density. However, when the prey density regularly alternates between low and high levels, the dormitive predator adapts through threshold‐based switching, whereas the perennially active predator cannot. When the intensity of prey density fluctuation is sufficiently high, the gain from the ability to adapt to these changes becomes large enough for the dormitive predator to out‐compete the perennially active one.

This effect helps explain our second counter‐intuitive finding: in the absence of abiotic variation, dormancy becomes increasingly adaptive as overall resource level increases, while being maladaptive at low resource levels. Many predator–prey interactions display the paradox of enrichment. Rather than stabilizing the system, enriched resources cause large‐amplitude population cycles due to prey oversupply followed by overpredation.^10^, ^12^, ^13^, ^43^ These population cycles ensure the regular alternation of prey density that dormitive predators can exploit. Without such cycles, dormancy goes to extinction. Furthermore, as the resource level increases, the population cycles grow larger in amplitude, conferring an even greater advantage to threshold‐induced dormancy.

In nature, large‐amplitude population cycles have been found to occur even when seasonal abiotic variation is minimal or insufficient to explain such fluctuations. For example, both Daphnia and plankton populations are known to fluctuate dramatically (over many orders of magnitude) during the growing season, with greater plankton fluctuations observed in enriched environments.^44^, ^45^ Such observations suggest that overpredation is at play, enabling the Parrondo's paradox mechanism described here. In addition, although it has been traditionally suggested that large‐amplitude population fluctuations are implausible because of the incumbent high extinction risk,^11^, ^12^, ^13^ this does not apply to dormitive predators that can withstand prey fluctuation due to the hardiness of their dormant forms. On the contrary, the larger the fluctuations, the more likely dormitive predators are to displace their perennially active competitors.

Several important predictions follow from the mechanism elucidated here. First, as long as large‐amplitude population fluctuations can occur, we should expect to see an increased proportion of dormitive predators (*z* > *y*) as resource levels (*K*) increase. Second, we should expect a higher prey threshold for the induction of dormancy, since η_c_ increases with *K*. Third, the key to the competitiveness and evolutionary stability of dormancy is severe fluctuations of the prey density. This can be caused by various factors other than an excess of resources, and so we should expect an increased presence of dormitive predators and higher dormancy thresholds as long as those fluctuations exist.

Beyond predator–prey ecology, our findings also suggest that Parrondo's paradox could have much broader relevance to biology than currently appreciated. In particular, our analysis of predator dormancy could be a useful framework for the analysis of closely related biological dynamics, such as dormancy in prey, host‐parasite coevolution, and any other system which exhibits self‐destructive dynamics akin to overpredation. For example, switches between lytic infection and lysogeny in bacteriophages could be considered a kind of “parasite dormancy,” where the bacteriophage alternates between dormancy through lysogeny and activity through host‐destroying lytic infection. Parrondo's paradox might then explain how this alternation emerges as a stable strategy. Subsequent theoretical and experimental research should explore and delineate the applicability of Parrondo's paradox to these areas.

## 4 Experimental Section

*Active‐Only and Dormitive‐Only Equilibria*: Expressions for the active‐only and dormitive‐only equilibria are first given, as they will be needed to derive further results. The dormitive‐only equilibrium $x_{z}^{*}=\left(p_{z}^{*},0,z_{1}^{*},z_{2}^{*}\right)$ is defined by setting $\dot{p},{\dot{z}}_{1},{\dot{z}}_{2}=0$, giving

(5a) $$d_{z_{1}}=k_{z_{1}}\mu \left(p\right)f_{z}\left(p\right)+{k^{\prime }}_{z_{2}}\left(1-\mu \left(p\right)\right)f_{z}\left(p\right)$$

(5b) $$z_{1}=\frac{r\left(1-p/K\right)p}{f_{z}\left(p\right)}$$

(5c) $$z_{2}=\frac{{k^{\prime }}_{z_{2}}}{\alpha }\left(1-\mu \left(p\right)\right)f_{z}\left(p\right)z_{1}$$

where the expression for $p_{z}^{*}$ is implicit in Equation (5a), and where $k\prime_{z_{2}}=\alpha k_{z_{2}}/\left(\alpha +d_{z_{2}}\right)$ is introduced for convenience. Note that $k\prime_{z_{2}}\leq k_{z_{2}}$ since $d_{z_{2}},\alpha \geq 0$. Now, the active predator is a special case of a dormitive predator where α = 0 (no “hatching”) and μ(*p*) = 1 (no switching). The active‐only equilibrium $x_{y}^{*}=\left(p_{y}^{*},y^{*},0,0\right)$ can thus be derived from Equations (5a) and (5b) by setting α = 0, μ(*p*) = 1 and replacing the parameters for *z* with those for *y*

(6a) $$d_{y}=\;\;\;k_{y}f_{y}\left(p\right)$$

(6b) $$y=\frac{r\left(1-p/K\right)p}{f_{y}\left(p\right)}$$

Similar to the dormitive case, the expression for $p_{y}^{*}$ is implicit in Equation (6a).

*Stability Conditions for Single‐Predator Equilibria*: Here the conditions given by Kuwamura et al. are restated^11^ for the stability of the above equilibria, as they will be needed for our own derivations. Additionally, they demarcate the regime in which large‐amplitude limit cycles emerge, since such cycles can only occur when there are no stable fixed‐point equilibria. Single‐predator stability was determined by ignoring the rate equations for the predator populations that were extinct at each equilibrium, then checking if the Jacobian of the reduced system was stable.

Let $J_{p,z_{1},z_{2}}$ be the 3 × 3 Jacobian matrix at $x_{z}^{*}$, with the *y* population removed from the system. Similarly, let *J*_*p*,*y*_ be the 2 × 2 Jacobian matrix at $x_{y}^{*}$, with *z*_1_ and *z*_2_ removed from the system. Kuwamura et al.^11^ showed that $J_{p,z_{1},z_{2}}$ is stable (has negative eigenvalues) when $K_{z}^{\mathrm{tc}}<K<K_{z}^{h}$, where $K_{z}^{\mathrm{tc}}$ is the transcritical bifurcation point, and $K_{z}^{h}$ is the Hopf bifurcation point. Correspondingly, the dormitive‐only equilibrium $x_{z}^{*}$ shows single predator‐stability when $K_{z}^{\mathrm{tc}}<K<K_{z}^{h}$. When α = 0, $K_{z}^{\mathrm{tc}}$ and *K* ^h^_*z*_ are given by

(7) $$K_{z}^{\mathrm{tc}}=p_{z}^{*},\;K_{z}^{h}=\frac{2p_{z}^{*}f_{z}\left(p_{z}^{*}\right)-{f^{\prime }}_{z}\left(p_{z}^{*}\right){\left(p_{z}^{*}\right)}^{2}}{f_{z}\left(p_{z}^{*}\right)-{f^{\prime }}_{z}\left(p_{z}^{*}\right)p_{z}^{*}}$$

In the general α ≥ 0 case, $K_{z}^{h}$ is lower‐bounded by the expression given above. For *J*_*p*, *y*_, it can again be noticed that the active predator is a special case of the dormitive predator with μ(*p*) = 1, α = 0. As such, *J*_*p*,*y*_ is stable when $K_{y}^{\mathrm{tc}}<K<K_{y}^{h}$, where $K_{y}^{\mathrm{tc}}$ and $K_{y}^{h}$ take on the same values as in Equation (7), except $p_{z}^{*}$ and *f*_*z*_(*p*) are replaced by $p_{y}^{*}$ and *f*_*y*_(*p*). Correspondingly, the active‐only equilibrium $x_{y}^{*}$ shows single predator‐stability when $K_{y}^{tc}<K<K_{y}^{h}$.

*Impossibility of Equilibrium Between Dormitive and Active Predators*: Under the assumptions of costly dormancy (*f*_*y*_(*p*) ≥ *f*_*z*_(*p*), $k_{y}\geq k_{z_{1}}>k_{z_{2}}$, $d_{y}\leq d_{z_{1}}$), it is straightforward to show the impossibility of coexistence of dormitive and active predators at an equilibrium $\left(p^{†},y^{†},z_{1}^{†},z_{2}^{†}\right)$ where $y^{†},z_{1}^{†},z_{2}^{†}>0$. Suppose such an equilibrium exists, implying ${\dot{z}}_{1},{\dot{z}}_{2},\dot{y}=0$. This gives us Equation (5a), as in the dormitive‐only equilibrium, as well as Equation (6a), as in the active‐only equilibrium. Now by assumption, $d_{z_{1}}\geq d_{y}$. Substituting $d_{z_{1}}$ from Equation (5a) and *d*_*y*_ from Equation (6a) gives

(8) $$(k_{z_{1}}-{k^{\prime }}_{z_{2}})\mu \left(p\right)f_{z}\left(p\right)\geq k_{y}f_{y}\left(p\right)-{k^{\prime }}_{z_{2}}f_{z}\left(p\right)$$

The above is combinedwith the assumption that *f*_*y*_(*p*) ≥ *f*_*z*_(*p*), giving

(9) $$\mu \left(p\right)\geq \frac{k_{y}-{k^{\prime }}_{z_{2}}}{k_{z_{1}}-{k^{\prime }}_{z_{2}}}$$

Since μ(*p*) < 1 and $k_{y}\geq \;\;k_{z_{1}}\geq \;\;k_{z_{2}}\geq \;\;{k^{\prime }}_{z_{2}}$, this inequality is a contradiction. Hence, an equilibrium $\left(p^{†},y^{†},z_{1}^{†},z_{2}^{†}\right)$ with $y^{†},z_{1}^{†},z_{2}^{†}>0$ is impossible.

*Instability of Dormitive‐Only Equilibrium to Active Invasion*: Here it is shown that the dormitive‐only equilibrium $x_{z}^{*}$ is unstable when the active population *y* is included in the system. The Jacobian at the dormitive‐only equilibrium, $J\left(x_{z}^{*}\right)$, can be written as


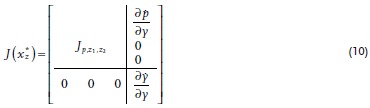


This is an upper‐triangular block matrix, so the eigenvalues of $J\left(x_{z}^{*}\right)$ are the union of the eigenvalues of the diagonal blocks, $J_{p,z_{1},z_{2}}$ and $\left[\frac{\partial \dot{y}}{\partial y}\right]$. As such, $\frac{\partial \dot{y}}{\partial y}$ is an eigenvalue of $J\left(x_{z}^{*}\right)$, and for the system to be unstable at $x_{z}^{*}$, it is enough to show that, $\frac{\partial \dot{y}}{\partial y}>0$, that is, the system is unstable to perturbations in *y*. Differentiating Equation (1b), we have $\frac{\partial \dot{y}}{\partial y}=k_{y}f_{y}\left(p\right)-d_{y}$. Combining the assumptions of costly dormancy with Equation (5a) then gives

(11) $$\begin{array}{l} k_{y}f_{y}\left(p\right)\geq k_{z_{1}}f_{z}\left(p\right)=k_{z_{1}}\mu \left(p\right)f_{z}\left(p\right)+k_{z_{1}}\left(1-\mu \left(p\right)\right)f_{z}\left(p\right)\\ \;\;\;\;\;>k_{z_{1}}\mu \left(p\right)f_{z}\left(p\right)+k_{z_{2}}^{\prime }\left(1-\mu \left(p\right)\right)f_{z}\left(p\right)=d_{z_{1}}\geq d_{y} \end{array}$$

It follows that $\frac{\partial \dot{y}}{\partial y}>0$, and hence the dormitive‐only equilibrium $x_{z}^{*}$ is unstable to active invasion.

*Stability of Active‐Only Equilibrium to Dormitive Invasion*: Now it is shown that if the active‐only equilibrium $x_{y}^{*}$ already exhibits single‐predator stability (*J*_*p*,*y*_ is stable), it remains stable when the dormitive populations *z*_1_, *z*_2_ are included in the system. The Jacobian at the active‐only equilibrium, $J\left(x_{y}^{*}\right)$, can be written as the block matrix

(12) $$J\left(y_{z}^{*}\right)=\left[\begin{array}{cc} J_{p,y} & D_{z_{1},z_{2}}^{\dot{p},\dot{y}}\\ 0 & J_{z_{1},z_{2}} \end{array}\right]$$

where $D_{z_{1},z_{2}}^{\dot{p},\dot{y}}$ contains the partial derivatives of $\dot{p}$, $\dot{y}$ with respect to *z*_1_, *z*_2_, and $J_{z_{1},z_{2}}$ is the Jacobian for the *z*_1_, *z*_2_ subsystem

(13) $$J_{z_{1},z_{2}}=\left[\begin{array}{c} k_{z_{1}}\mu \left(p\right)f_{z}\left(p\right)-d_{z_{1}}\\ k_{z_{2}}\left(1-\mu \left(p\right)\right)f_{z}\left(p\right) \end{array}\begin{array}{c} \alpha \\ -\alpha -d_{z_{2}} \end{array}\right]$$

$J\left(x_{y}^{*}\right)$ is an upper‐triangular block matrix, so the eigenvalues of $J\left(x_{y}^{*}\right)$ are the combined eigenvalues of *J*_*p*,*y*_ and $J_{z_{1},z_{2}}$. Hence, if *J*_*p*,*y*_ is already stable, it follows that the active‐only equilibrium $x_{y}^{*}$ is stable when $J_{z_{1},z_{2}}$ is stable (the system is stable to perturbations in *z*_1_ and *z*_2_). Since $J_{z_{1},z_{2}}$ is a 2 × 2 matrix, it is stable if $\mathrm{tr}\left(J_{z_{1},z_{2}}\right)<0$ and $\det\left(J_{z_{1},z_{2}}\right)>0$. To see that $\mathrm{tr}\left(J_{z_{1},z_{2}}\right)<0$, first note that $-\alpha -d_{z_{2}}<\;\;0$. Furthermore, by the costly dormancy assumptions and Equation (6a), we have

(14) $$k_{z_{1}}\mu \left(p\right)f_{z}\left(p\right)<k_{y}f_{y}\left(p\right)=d_{y}\leq \;\;d_{z_{1}}$$

Hence $k_{z_{1}}\mu \left(p\right)f_{z}\left(p\right)-d_{z_{1}}<\;\;0$, which implies $\mathrm{tr}\left(J_{z_{1},z_{2}}\right)<0$. Now $\det\left(J_{z_{1},z_{2}}\right)$ can be expressed as

(15) $$\begin{array}{l} \det\left(J_{z_{1},z_{2}}\right)=\alpha \left[d_{z_{1}}-\;\;k_{z_{1}}\mu \left(p\right)f_{z}\left(p\right)-k_{z_{2}}\left(1-\mu \left(p\right)\right)f_{z}\left(p\right)\right]\\ \;\;\;\;\;\;\;\;\;\;\;\;\;\;+\;d_{z_{2}}\left[d_{z_{1}}-\;\;k_{z_{1}}\mu \left(p\right)f_{z}\left(p\right)\right] \end{array}$$

The second term is positive since $k_{z_{1}}\mu \left(p\right)f_{z}\left(p\right)-d_{z_{1}}<\;\;0$, and the first term is positive because

(16) $$d_{z_{1}}\geq d_{y}=k_{y}f_{y}\left(p\right)\geq k_{z_{1}}f_{z}\left(p\right)>k_{z_{1}}\mu \left(p\right)f_{z}\left(p\right)+k_{z_{2}}\left(1-\mu \left(p\right)\right)f_{z}\left(p\right)$$

It follows that $\det\left(J_{z_{1},z_{2}}\right)>0$ and $J_{z_{1},z_{2}}$ is stable. As such, the active‐only equilibrium $x_{y}^{*}$ is stable to dormitive invasion.

*Impossibility of Equilibrium with Mixed Dormancy Thresholds*: In the case of two dormitive populations *y* and *z* with differing dormancy thresholds η_*y*_ ≠ η_*z*_, it can also be shown that equilibrium coexistence is impossible. Suppose that such an equilibrium exists, that is, $\dot{p},{\dot{y}}_{1},{\dot{y}}_{2},{\dot{z}}_{1},{\dot{z}}_{2}=0$, with *p*, *y*_1_, *y*_2_, *z*_1_, *z*_2_ > 0. Setting Equations (4b) and (4c) to zero and rearranging, the following equations are obtained

(17) $$\begin{array}{l} d_{1}=\left[{k^{\prime }}_{2}+\left(k_{1}-{k^{\prime }}_{2}\right)\mu_{y}\left(p\right)\right]f\left(p\right)\\ d_{1}=\left[{k^{\prime }}_{2}+\left(k_{1}-{k^{\prime }}_{2}\right)\mu_{z}\left(p\right)\right]f\left(p\right) \end{array}$$

where the placeholder *q* is substituted for both *y* and *z*, and ${k^{\prime }}_{2}=\alpha k_{2}/\left(\alpha +d_{2}\right)$ is also introduced for convenience. However, this leads to a contradiction, because when η_*y*_ ≠ η_*z*_, then μ_*y*_(*p*) ≠ μ_*z*_(*p*) for a given *p*, and so the above two equations cannot hold simultaneously. It follows that two populations with differing dormancy thresholds cannot coexist in equilibrium.

*Determining Dominance and Extinction*: Here the numerical methods are detailed for distinguishing cases of species dominance and extinction. For the active–dormitive model (Equations (1a)–(1d)), numerical simulations were run up to a maximum time of *T* = 3000, and the time‐averaged population densities of both predator species were calculated for *t* ∈ [T*, *T*] = [2600, 3000] as $\bar{q}=\int_{T^{*}}^{T}q\left(t\right)\;dt/\left(T-T^{*}\right)$ where *q* ∈ {*y*, *z*}. The integration was performed numerically using a global adaptive scheme^46^, ^47^ robust to rapidly oscillating functions. If the time‐averaged population density of a species was below a threshold Λ = 10^−3^, that species was considered extinct; if neither species was found to be extinct, the species with the higher time‐averaged population density was considered dominant. The values of *T* and T* were verified to be sufficiently large for convergence of the population densities to either equilibrium or limit cycles, for all parameter spaces presented in Figure 5.

The determination of dominance and extinction in the dormitive–dormitive model (Equations (4a)–(4c)) was complicated by the extremely slow convergence of population densities for certain parameter combinations, for instance when η_*y*_ ≪ 0 and η_*z*_ ≪ 0, and when in close proximity to the critical level η_c_. The slow convergence required the numerical simulations to be run to large *T* > 10^5^ in order for determination on species dominance and extinction to be performed accurately. A three‐stage refinement process was hence utilized to generate the phase diagrams in Figure 6. First, a base sweep across the η_*y*_–η_*z*_ parameter space was performed to a maximum time of *T* = 2 × 10^4^, exploiting the symmetry along the η_*y*_ = η_*z*_ axis. Next, all nonextinct cases were refined up to a larger maximum time of *T* = 10^5^. Lastly, all samples of η_*y*_ < 0 and η_*z*_ < 0 and in the neighborhood of η_c_, regardless of extinction or dominance status, were refined to a large maximum time of *T* = 5 × 10^5^. The computation of η_c_ for each value of *K* is detailed in Section 4. This procedure enabled good accuracy with manageable computational cost.

Similar to the active–dormitive model, dominance and extinction were distinguished by comparing the time‐averaged population density over the last Δ*T* = 200 time units of each simulation, using an extinction threshold of Λ = 10^−1^. If neither species was found to be extinct, the eventual result was extrapolated by determining whether the system had converged to a limit cycle, or was on an exponential decay trajectory. This was done by fitting the numerical solution *q*(*t*) over the last Δ*T* time units to an exponential function $\hat{q}\left(t\right)=A_{1}\exp\left(-A_{2}t\right)+A_{3}$ via Levenberg–Marquardt nonlinear regression.^48^, ^49^, ^50^ If the sum of squared residuals was above a threshold Ξ = 10, the population densities were considered to be oscillatory, and whichever species with the higher time‐averaged density $\bar{q}$ was considered dominant. Otherwise the populations were considered to be converging toward steady‐state, and the species with lower $\bar{q}$ was extrapolated to go extinct.

*Solving for the Critical Dormancy Threshold*: The critical dormancy switching threshold η_c_ as presented in Figures 6 and 7 was computed via a bisection method. The method begins with lower and upper search bounds η^−^ and η^+^, set to cover the η_*y*_–η_*z*_ parameter spaces in Figure 6. The interval [η^−^, η^+^] was divided into a set of *n*_η_ = 11 sample points *S* = {η^−^ + *i*Δη: 0 ⩽ *i* ⩽ *n*_η_ ‐ 1, Dη = (η^+^ − η^−^)/(*n*_η_ − 1)}, and the long‐run outcome of the dormitive–dormitive model was evaluated for each (η_*y*_, η_*z*_) = (*s* − ∈, *s* + ∈) where *s* ∈ *S* and ∈ = 0.05 is a small offset constant.

These numerical simulations were run up to a maximum time of *T* = 2 × 10^4^, and whichever predator species with higher time‐averaged population density for *t* ∈ [*T* − 200, *T*] was considered to be winning, calculated using the same approach detailed earlier. New bounds *s* ^−^ and *s* ^+^ are identified as the maximal and minimal *s* ∈ *S* sandwiching the transition between winning and losing outcomes, and a new iteration begins with η^±^ = *s* ^±^. A total of *N* = 10 iterations were utilized in the generation of Figures 6 and 7, yielding an error of η_c_ on the order of 10^−9^.

## Conflict of Interest

The authors declare no conflict of interest.

## Supporting information

Supporting Information
