# Supplementary material for: Predator Dormancy is a Stable Adaptive Strategy due to Parrondo's Paradox
Source: Adv Sci (Weinh). 2019 Dec 12;7(3):1901559. doi: 10.1002/advs.201901559 (PMC7001654; doi:10.1002/advs.201901559)
Supplement: Supplementary file 1 — Supporting Information [file ADVS-7-1901559-s001.pdf]

Copyright WILEY-VCH Verlag GmbH & Co. KGaA, 69469 Weinheim, Germany, 2019.

ADVANCED SCIENCE
Open Access

# Supporting Information

for *Adv. Sci.,* DOI: 10.1002/advs.201901559

# Predator Dormancy is a Stable Adaptive Strategy due to Parrondo's Paradox

*Zhi-Xuan Tan, Jin Ming Koh, Eugene V. Koonin, and Kang Hao Cheong**

Supplementary Information

# Predator dormancy is a stable adaptive strategy due to Parrondo's paradox

Zhi-Xuan Tan[1], Jin Ming Koh[1], Eugene V. Koonin[2], and Kang Hao Cheong[1,3,*]

[1]Science and Math Cluster, Singapore University of Technology and Design (SUTD), S487372, Singapore
[2]National Center for Biotechnology Information, National Library of Medicine, National Institutes of Health, Bethesda, MD 20894, USA
[3]SUTD-Massachusetts Institute of Technology International Design Centre, S487372, Singapore
[*]Corresponding author: Kang Hao Cheong (kanghao_cheong@sutd.edu.sg)

## Supplementary Discussion

As noted in the main paper, predator dormancy can remain advantageous even when the active predator is more proficient than the dormitive predator. This can be seen from the phase diagrams in Figure 1. In particular, the blue regions above the dashed lines in Figures 1(a)–(c) show how the dormitive predator can out-compete an active predator with higher growth efficiency ($k_y > k_{z_1} = 0.50$) as long as $K$ or $k_{z_2}$ are sufficiently high, or if $d_{z_2}$ is sufficiently low. Similarly, the blue regions below the dashed lines in Figures 1(d)–(f) show how the dormitive predator can out-compete an active predator with lower death rate ($d_y < d_{z_1} = 0.25$) as long as $K$ or $k_{z_2}$ are sufficiently high, or if $d_{z_2}$ is sufficiently low. Higher $K$ increases the sharpness of oscillations in the prey population, higher $k_{z_2}$ allows the dormant population $z_2$ to grow more efficiently, and lower $d_{z_2}$ means a slower attrition rate of the dormant population, each of which make dormancy more advantageous for the dormitive predator.

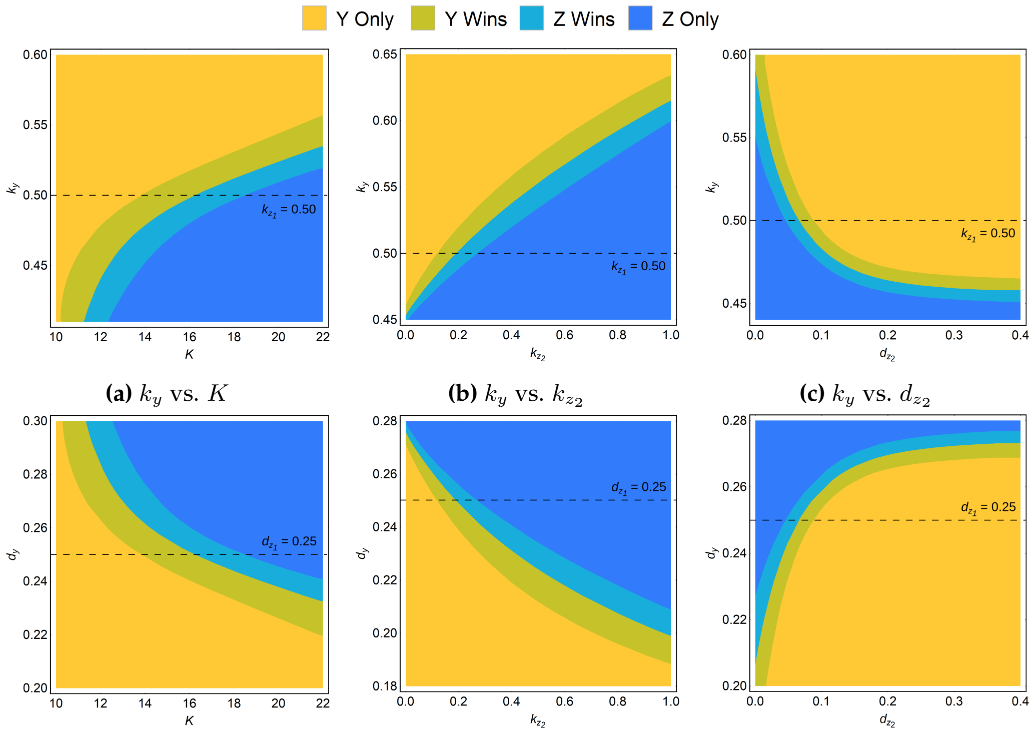


**(a)** $k_y$ vs. $K$ **(b)** $k_y$ vs. $k_{z_2}$ **(c)** $k_y$ vs. $d_{z_2}$

**(d)** $d_y$ vs. $K$ **(e)** $d_y$ vs. $k_{z_2}$ **(f)** $d_y$ vs. $d_{z_2}$

**Figure 1:** Phase diagrams showing the parameter regimes where only the active predator survives ($y$ only), the active predator dominates the dormitive predator ($y$ wins), the dormitive predator dominates the active predator ($z$ wins), or only the dormitive predator survives ($z$ only). The dormitive predator has a stronger competitive advantage as $K$ increases, $k_{z_2}$ increases, or $d_{z_2}$ decreases, allowing it to outcompete active predators with higher growth efficiency ($k_y > k_{z_1}$), as shown in panels (a)–(c), or lower death rate ($d_y < d_{z_1}$), as shown in panels (d)–(f). Default parameter values as in Table 1 of the main paper, unless stated otherwise; simulation methodology is identical to that detailed in the main paper.
